# Phase Behavior and Composition Distribution of Multiphase Hydrocarbon Binary Mixtures in Heterogeneous Nanopores: A Molecular Dynamics Simulation Study

**DOI:** 10.3390/nano11092431

**Published:** 2021-09-18

**Authors:** Deraldo de Carvalho Jacobina de Andrade, Bahareh Nojabaei

**Affiliations:** Department of Mining and Minerals Engineering, Virginia Polytechnic Institute and State University, Blacksburg, VA 24061, USA; deraldo@vt.edu

**Keywords:** nanopore, confinement effect, porous media, shale, phase behavior, hydrocarbon mixture, molecular dynamics

## Abstract

In this study, molecular dynamics (MD) simulation is used to investigate the phase behavior and composition distribution of an ethane/heptane binary mixture in heterogeneous oil-wet graphite nanopores with pore size distribution. The pore network system consists of two different setups of connected bulk and a 5-nm pore in the middle; and the bulk connected to 5-nm and 2-nm pores. Our results show that nanopore confinement influences the phase equilibrium of the multicomponent hydrocarbon mixtures and this effect is stronger for smaller pores. We recognized multiple adsorbed layers of hydrocarbon molecules near the pore surface. However, for smaller pores, adsorption is dominant so that, for the 2-nm pore, most of the hydrocarbon molecules are in the adsorbed phase. The MD simulation results revealed that the overall composition of the hydrocarbon mixture is a function of pore size. This has major implications for macro-scale unconventional reservoir simulation, as it suggests that heterogenous shale nanopores would host fluids with different compositions depending on the pore size. The results of this paper suggest that modifications should be made to the calculation of overall composition of reservoir fluids in shale nanopores, as using only one overall composition for the entire heterogenous reservoir can result in significant error in recovery estimations.

## 1. Introduction

The depletion of conventional oil and gas reservoirs directed oil and gas companies and researchers to pursue alternatives in the formations that, in the past, were considered economically unrecoverable. The consequential advance in drilling and completion technologies, mainly horizontal drilling and hydraulic fracturing, enabled the exploration of these “unconventional” reserves. Unconventional reservoirs characteristics include a low porosity, not always the case, but mainly a low permeability medium. Some examples are source rocks, and coal bed methane. The former, as the name suggests, are rocks, most commonly shale, which generates petroleum by the maturation of the organic matter, such as kerogen, present in them. The extremely high pressures in which these reservoirs are normally encountered, along with the fine particles deposition environment they were generated at, resulted in pores in the nanometer scale, in contrast to the millimeter or micrometer scale for conventional reservoirs. The complicated nanopore rock structure, which is reported to influence phase behavior and transport properties of fluids [1,2], can affect standard reservoir simulation equations, such as oil in place (OIP) predictions [3].

**Figure 1:**
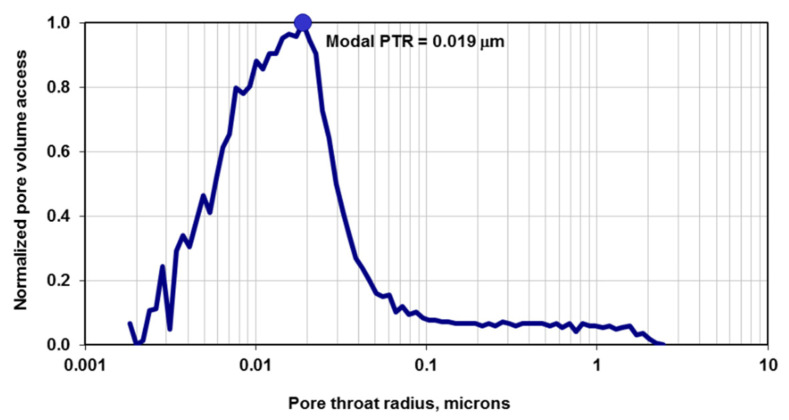
Typical measured pore throat radius (PTR) of Bakken shale rock from high-pressure mercury-air capillary pressure experiments [3]. This plot indicates that the majority of the pores have PTR of 19 nm, but the sample is heterogenous in terms of pore size (ranging from 2 nm to 2 microns).

Macroscopic modeling methods are not able to provide answers to the questions regarding the hydrocarbon fluid phase behavior and transport properties, as well as rock–fluid interactions in the shale nanopores. Destructive high-resolution three-dimensional imaging techniques, such as the focused ion beam scanning electron microscope (FIB-SEM), do not provide a useful answer either, since they only work with dry rock samples and in vacuum conditions, and visualization of multiphase fluid coexistence in nanopores at reservoir pressure and temperature is not possible. High-resolution X-ray computed tomography (CT) non-destructive imaging tools might be an alternative option to investigate fluid properties in nanopores; however, the highest resolution for these tools can only be 50 nm. Shale and tight rocks are associated with nanopores as small as 2–3 nanometers (as shown for a Bakken rock sample in Figure 1 [3]), and using such imaging tools would not provide enough information to analyze multiphase coexistence in such small pores, as it is impossible to visualize multiple phases in such small pores.

Molecular scale simulation is likely the most accurate and suitable method to investigate the interactions of rock (pore wall) surface, and oil and gas in nanopores (less than 50 nm pore size). Among molecular simulation methods, molecular dynamics (MD) is a powerful tool, which can simulate the behavior of a system in molecular scale by solving the Newton’s equations of motion for each individual molecule to find the new position of that molecule with respect to others. To investigate rock–fluid interactions using MD simulation, the rock and the fluids in nanopores should be represented by molecules and structures that mimic these natural systems as closely as possible.

Even though reservoir fluids and pores interactions have been studied using MD simulation, these models used a simplistic pore crystal structure, as well as pore geometry or fluid composition. Welch et al. used graphite to model the organic pore matrix that is common to these rocks. They observed the retrograde condensation of a 70/30 weight % ethane/heptane mixture at bulk phase, but they did not investigate phase transition in the nanopore [4]. Zhong et al. analyzed the adsorption mechanism and preference on a silica surface, but they only studied these phenomena on the surface of a single slab, so they underestimated the effect of pore vertices and corners [5]. Ambrose et al. calculated the mass density increase on the surface of a graphite nanopore, while they used a slit pore model that did not account for the effect of a full-walled pore [6]. Wu et al. detected the effect of wettability on hydrocarbon accumulation on an organic shale pore surface, but they only used a single surface slab for their study [7]. Among the very first works to investigate the effect of nanopore confinement on thermodynamic properties in detail is the study by Pitakbunkate et al., who used two graphene slabs to represent kerogen to examine the effect of confinement on critical properties of methane and ethane, and to estimate density and saturation pressure changes of the methane/ethane mixture in nanopore-confined spaces ranging in size from 1 to 10 nm [8]. Jin and Nasrabadi also used Grand Canonical Monte Carlo (GCMC) simulation to analyze the phase behavior of selected binary and ternary hydrocarbon mixtures in a 4-nm slit graphene pore and recognized a shift in the critical point composition. They also noticed an uneven density profile of the confined fluid with heavier molecules accumulated near the pore surface [9]. In another research work, they investigated the phase behavior of a C_1_/C_3_/nC_5_ mixture in organic and inorganic slit and cylindrical pores [10]. They made binodal curves of the ternary mixtures on ternary diagrams in the bulk and nanopore-confined space with different sizes, and concluded that nanopore confinement effect of the cylindrical pores was stronger.

Shale rock is naturally heterogeneous and consists of a distribution of pore sizes. Pore size distribution of the heterogenous rock can make the nanopore confinement effects even more complicated. Fracture–matrix systems are also considered as heterogenous porous media with different pore sizes and permeabilities. We envision that the wide range of pore size distribution in unconventional reservoirs impacts the composition of reservoir fluid, which in turn affects the original hydrocarbon in place and recovery mechanisms. Jin et al. used multiple cylindrical pores with different sizes in their molecular modeling to account for pore size distribution; however, in their model, pores do not communicate [11]. Neither of the research works which used molecular scale analysis to study the phase behavior of multicomponent mixtures in nano-confined space considered connected pores with different sizes.

Here, a molecular dynamics study of nano-confinement effect on hydrocarbon fluid phase and concentration distribution in organic shale rock with pore size distribution is presented. The pore is modeled by a fully atomistic squared graphite pore that satisfies all the requirements of the force field in use. This pore accounts for the total effect of wall surfaces and vertices effects. The model has the capacity to simulate more oil-wet behavior by manipulation of radicals on the surface. The hydrocarbon fluid mixture is composed of a 70/30 weight % of ethane and heptane, respectively. The system is subjected to reservoir temperatures and pressures, and the outputs are analyzed by density and compositional variation through the simulation box to observe phase behavior. Simulations include hydrocarbon fluid mixture at bulk and nanopore conditions, and hydrocarbon migration and accumulation in the bulk and the connected nanopores with different sizes. The simulation results are analyzed in a molecular scale, and our understanding from the micro-scale is expanded to the core and field scale practical petroleum engineering applications.

## 2. Computational Methods

In this section, all the simulation models, parameters, and programs used in this study are presented. First, we discuss how molecular dynamics (MD) simulations are performed. MD is the time-dependent behavior of molecules under a force field, while the molecules motion inside the simulated box obeys the Newton’s second law of motion. The velocity and location of the molecules at each time step are then influenced by the forces that are applied to each atom in a molecule. The forces applied to each atom in a MD simulation are calculated as the negative gradient of the intermolecular interaction potential of that atom for each time step during the simulation, and are essentially based on the sum of non-bonded and bonded interactions. Each atom receives a specific value for the above-mentioned parameters, and they differ from force field to force field. The force fields are acquired by either ab-initio molecular dynamics simulations, or experiments [12,13]. In the next sections, the force field choice, simulation models, and numerical experiment setup are exhibited.

### 2.1. Molecular Simulation Details

All the simulations in this study are performed utilizing GROMACS simulation tool (v.2020.1) [14,15]. The OPLS-AA force field [16] is used to represent both pore and hydrocarbon molecules since its parameters were acquired from ab-initio calculations and tuned to relevant experimental thermodynamics properties measurements of multiple organic molecules in through fitting processes. Water is represented by the extended simple point charge potential (SPC/E) since it accurately approximated the mass density of water [13]. To calculate van der Waals interactions, Verlet algorithm [17] with a 1.5-nm cut-off distance (the range in which the algorithm stops searching for nearby atoms to be included in the calculation) is applied. For long-range electrostatics interactions, past cut-off distance, the particle mesh Ewald (PME) algorithm [18] is employed. NVT ensemble (constant number of molecules, constant volume, and constant temperature) is used in our simulation. NVT is appropriate here in the sense that we can mimic the actual thermodynamic process in a closed system with constant volume and temperature, which is very close to what occurs in the rock porous media of petroleum reservoirs. To set pressure for this system, macroscopic thermodynamics relations of pressure, density, volume, temperature, and number of molecules are used. We calculate the number of molecules in the simulation box to provide the required pressure before the start of MD simulation. After the simulation is performed, we record pressure as an output and compare it with the initially calculated pressure to make sure that the estimated pressure is correct. If it is not, we will repeat the simulation with adjusted number of molecules. The simulation runs take at least 5 ns (or longer if needed, to assure the system is at equilibrium), which results in a total of 2,500,000 time steps. We make sure that this time is long enough for the system to equilibrate trough tacking the simulation results. The output of the simulations is analyzed through using our in-house FORTRAN codes, and the visualization is performed by VMD [19].

### 2.2. Shale Pore Model Details

**Figure 2:**
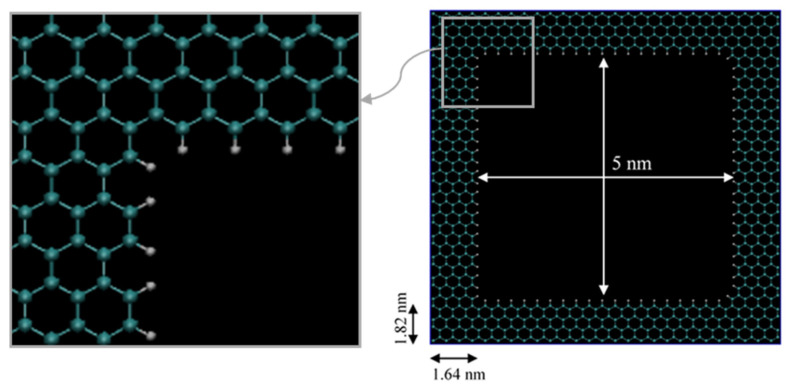
Graphene sheet structure. The dangling bonds are connected to (-H). Cyan and white are carbon and hydrogen atoms, respectively.

Only a few previous studies have implemented pores with irregular cross sections (non-circular and not a slit-pore) [20]. When they did, the pore walls connections at the vertices are unnatural since the force field they used did not account for these bonds on its parametrization. Here, we present a simulation of a fully atomistic squared organic shale pore that fully satisfies all the requirements of the force field in use, and that correctly describes bonds at the vertices. The force field (OPLS-AA) in use here is parametrized for organic molecules, and the pore model used in this study is simply a carbon-based structure, graphene, so the bonds, angles, and dihedrals as well as the interactions with other molecules are included in the force field. The fluid in the pores is a mixture of 70 weight % ethane and 30 weight % heptane. The organic shale pore is based on a graphite rock. Graphite essentially consists of compact layers of graphene, which have an average of 0.335 nm space in between. The graphene sheet model can be seen in Figure 2. This model consists of a layer of sp2-bonded carbons arranged on a hexagonal lattice, much like a honey bee panel. Then, an in-house FORTRAN code is utilized to remove all the atoms and bonds in the desired radius from the center of the sheet. The carbon dangling bonds are then connected to hydrogen (-H) (Figure 2), creating a desired more oil-wet surface.

**Figure 3:**
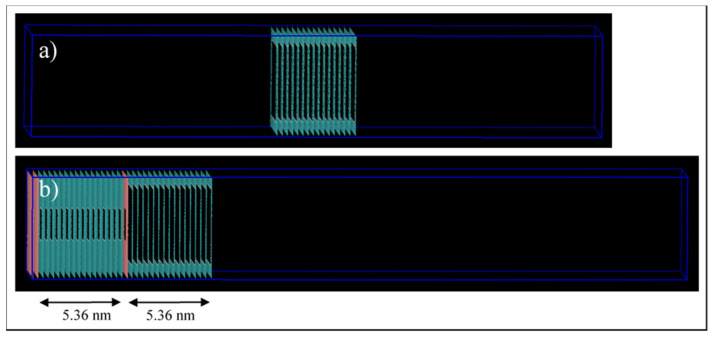
Simulation box for the pore models. (**a**) Single pore, and (**b**) connected 5-nm to 2-nm pores.

Two pore models are implemented here, the first one, shown in Figure 3a, consists of 16 stacked sheets of graphene, 0.335 nm apart, which results in a 5.36 nm long pore. The pore model has a 5 × 5 nm^2^ squared cross-section. The pore walls are 1.64 nm and 1.82 nm in x and y directions, respectively, which is higher than the 1.5-nm cut-off distance. The pore is then placed at the middle of the simulation box, and a total volume of 6.64 × 6.82 × 30 nm^3^ (15 nm on each side) is designated to be filled by the fluid molecules. The second one, shown in Figure 3b, consists of two connected pores. This pore model is formed through incorporating the design of the first model and connecting it to a 2 × 2 nm^2^ squared cross-section pore, which is constructed similarly to the 5-nm pore. The connected pores are then placed at the left side of the simulation box and the 2-nm pore end that interacts with the bulk phase is blocked. We do this to ensure that the molecules interacting with this pore can only come from the 5-nm pore. A total volume of 6.64 × 6.82 × 30 nm^3^ is left as a void space to be filled by fluid molecules.

## 3. Results and Discussion

In this study, we analyze both the phase behavior and mass transport of hydrocarbon molecules in the bulk and in nanopores. Specifically, we are interested to characterize and compare the phase equilibrium of hydrocarbon mixtures at multiple pressures and temperatures in the bulk and in nano-confined spaces. We also examine the hypothesis that pore size distribution, e.g., two connected pores with different sizes which communicate with the bulk, can result in selective molecules movement and different composition distribution in different pores.

### 3.1. Mass Density and Phase Behavior Analyses in Bulk and Nanopore

Here, the fluid mass density is monitored both in the bulk and nano-confined spaces. The idea is to observe the ability of MD simulations to replicate phase characteristics in the bulk, and to account for the nano-confinement effect when a pore is introduced to the system. The fluids are tested in three temperature and pressure conditions, where condition 1 (C1) is the gas phase in the bulk, condition 2 (C2) is the two-phase in the bulk, and condition 3 (C3) is the liquid phase in the bulk. We start with pure ethane and heptane molecules and extend the simulations to model phase behavior and transport of mixtures. The simulations for the ethane–heptane mixture are conducted in the bulk, bulk communicating with the 5-nm pore, and bulk communicating with two connected pores (2 and 5 nm).

#### 3.1.1. Pure Ethane

Ethane is a light hydrocarbon encountered in most light oil or gas reservoirs. Since unconventional reservoirs have high temperatures and pressures, this molecule has an even higher chance to appear due to cracking of heavier hydrocarbons or kerogen molecules. In this section, three different pressure and temperature conditions are studied: C1: 8 °F, 14.7 psi (a gas phase in bulk), C2: 68 °F, 546.21 psia (saturation temperature and pressure of ethane in bulk, a two-phase in bulk), and C3: 68 °F, 4000 psi (a liquid phase in bulk).

Under these conditions, the bulk mass densities are 1.26, 86.39, 447.73 kg/m^3^ for C1, C2, and, C3, respectively. The mass densities, under these pressures and temperatures, are acquired from the NIST webbook [21].

**Figure 4:**
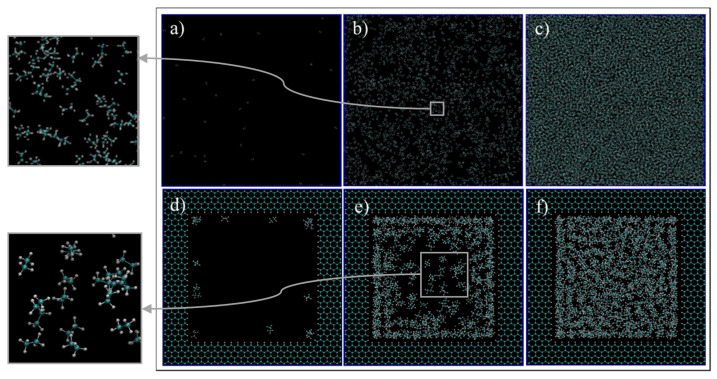
Ethane molecules simulation in bulk (**a**–**c**) and in the nano-pore (**d**–**f**) at C1 (**a**,**d**), C2 (**b**,**e**), and C3 (**c**,**f**). C1, C2, and C3 refer to temperature and pressure conditions of 68 °F and 14.7 psia, 68 °F and 546.21 psia, and 68 °F, 4000 psia, respectively. Cyan and white spheres are carbon and hydrogen atoms, respectively.

**Figure 5:**
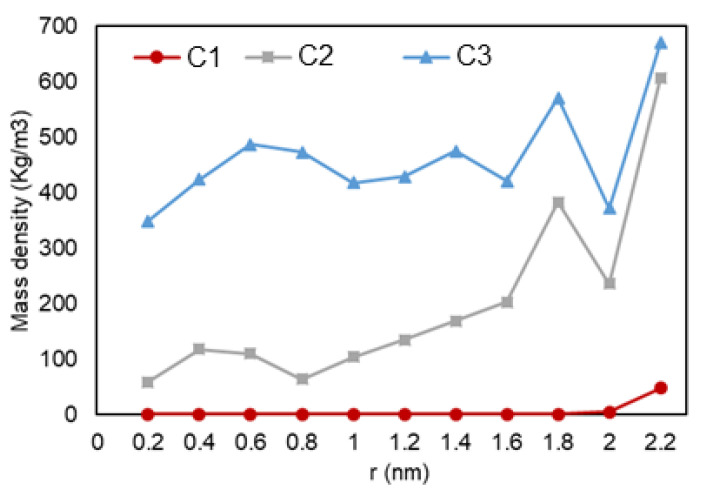
Ethane mass density vs. radial position in C1 (gas phase in bulk), C2 (two-phase in bulk), and C3 (liquid phase in bulk) conditions.

First, we qualitatively analyze the results of the simulations given in Figure 4. For C1 (Figure 4a,d, 68 °F, 14.7 psia), the simulation exhibits spaced out molecules, as expected for the gas phase. However, for the confined pore space, all of the molecules inside the pore are accumulated adjacent to the walls, and the majority of them are close to the vertices (Figure 4d). This later observation is justifiable as there is a higher concentration of pore carbon atoms at the vertices, which then translates to higher attraction of ethane molecules by van der Waals interaction, of the type London dispersion, since both pore and ethane molecules are non-polar. For C2 (Figure 4b,e, 68 °F, 546.21 psia), the bulk simulation shows a mixed pattern of accumulated molecules and void spaces, which implies that there are gas and liquid phases in our system. This trend is even more present in the confined pore space; near the wall, there is high accumulations of ethane, while at the center, the void space is predominant (Figure 4e). This observation implies that, due to nano-confinement, there is a presence of a liquid phase near the pore walls, while a gas phase is present inside the pore. This observation can be justified and quantified in Figure 5, where the mass density is closer in value to the bulk gas density in the center of the pore, while near the pore wall, the density is comparable to the bulk liquid density. For C3 (Figure 4c,f, 68 °F, 4000 psia), there is almost no void space, so all of the molecules are compact, which is indeed the characteristics of the liquid phase. A similar trend can be observed in the pore-confined space, but again higher accumulations of molecules can be observed near the pore walls and the distribution of molecules is not as homogenous as of the bulk phase, even at the center of the pore (Figure 4f). It seems like the fluid–solid interactions influence the distribution of ethane molecules and results in multiple adsorption layers near the pore surface and clusters near the pore center.

**Figure 6:**
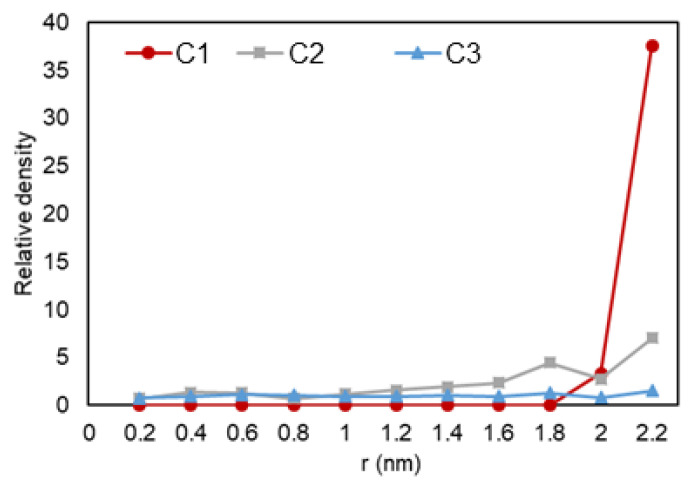
Ethane relative density (mass density inside the pore over the density of the phase in bulk conditions) vs. radial position in C1 (gas phase in bulk), C2 (two-phase in bulk), and C3 (liquid phase in bulk) conditions.

The density over the pore radius was calculated by developing and using an in-house FORTRAN code that identified the number of atoms alongside the area from the center to the pore walls. As shown in Figure 5, it can be observed that there is a lower density region at the center of the pore, while there is a high rise in the number of ethane molecules towards the pore walls. The highest density is observed at the walls, which is translated to adsorption. Another highlight is the multiple peaks (two for C2 and three for C3) in the graph, which can represent a second (and third) adsorption layer, a common trend that is also observed in experiments and prior simulations. In Figure 6, relative density vs. radial position is analyzed for the three cases, and it can be observed that ethane molecules at C1 condition (red) is the one mostly affected by nano-confinement, while the C2 system (blue) is the least affected. Note that the relative density here is not calculated through normalizing the density with the density at the pore center (r = 0), so the relative density profile does not regress to one in the middle of the pore where r approaches zero.

#### 3.1.2. Pure Heptane

Compared to ethane, heptane is a heavier molecule that is mostly present in the liquid fraction of reservoir oils. This component is encountered in a gas phase at a fewer range of pressures and temperatures in contrast to ethane. Furthermore, in order to simulate it in a gas phase, the temperature of the simulations had to be set at 248 °F. Even though it is an elevated temperature, this setup is commonly found in unconventional reservoirs that are located at greater depths.

**Figure 7:**
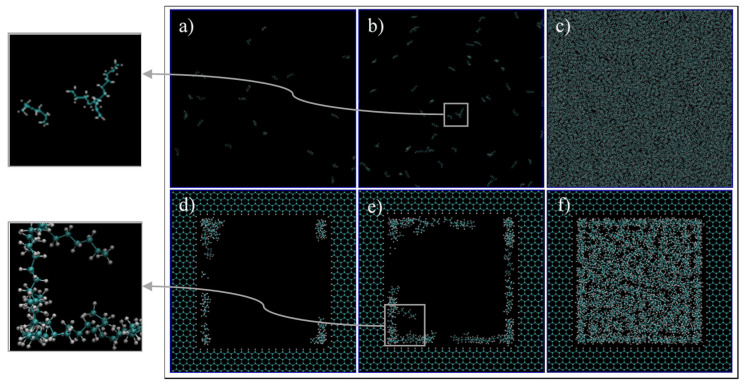
Heptane molecules simulation in bulk (**a**–**c**) and pore medium (**d**–**f**) at C1 (**a**,**d**), C2 (**b**,**e**), and C3 (**c**,**f**) condition. C1, C2, and C3 refer to temperature and pressure of 248 °F, 14.7 psia, 248 °F, 54.17 psia, 248 °F, 4000 psia, respectively. Cyan and white spheres are carbon and hydrogen atoms, respectively.

Again, three scenarios or conditions were studied in this section that would result in a gas phase, a two-phase (gas/liquid), and a liquid phase in the bulk, and the pictures of the molecules at the end of simulations are shown in Figure 7. For C1 (Figure 7a, 248 °F, 14.7 psia, and bulk density of 3.25 kg/m^3^), the simulation exhibits dispersed heptane molecules that move freely apart from each other, as expected for a gas phase. However, when a pore is introduced (Figure 7d), heptane molecules migrated to the pores walls and their movement is restricted to this confined region, especially to the corners of the graphene pore. For C2, which is expected to result in the two-phase mixture in bulk (Figure 7b, 248 °F, 54.17 psia, bulk density of 6.12 kg/m^3^), heptane molecules are more agglomerated, and lumps of three molecules can be seen moving in conjunction. Even though this cannot be considered a liquid phase, it represents a more liquid-like behavior. At nano-confined conditions (Figure 7e), the heptane molecules coat the pore surface, and again a higher accumulation of these molecules can be found at the corners of the pore. For C3 (Figure 7c, 248 °F, 4000 psia, bulk density of 635.79 kg/m^3^), a massive agglomeration of heptane molecules is encountered at the bulk, and the molecules have limited space to move around since there is almost no void space. This representation is expected for liquid phases. When a pore is introduced (Figure 7d), a similar trend is observed, but in this case a higher concentration of molecules is encountered near the walls. Similar to ethane, the molecules preferentially are located near the walls, which represents an adsorption behavior.

**Figure 8:**
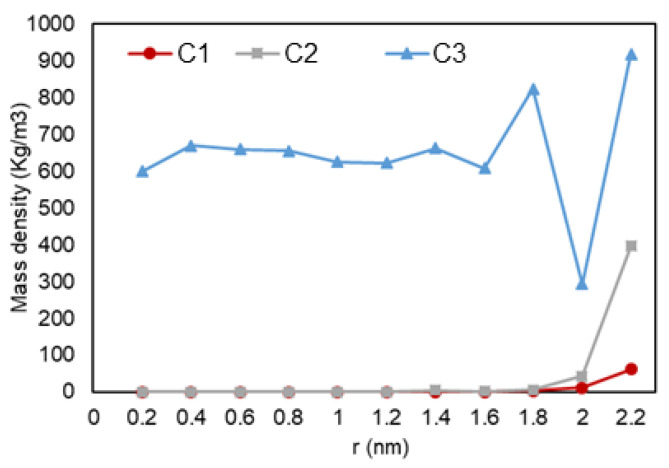
Heptane mass density vs. radial position in C1 (gas phase in bulk), C2 (two-phase in bulk), and C3 (liquid phase in bulk) conditions.

**Figure 9:**
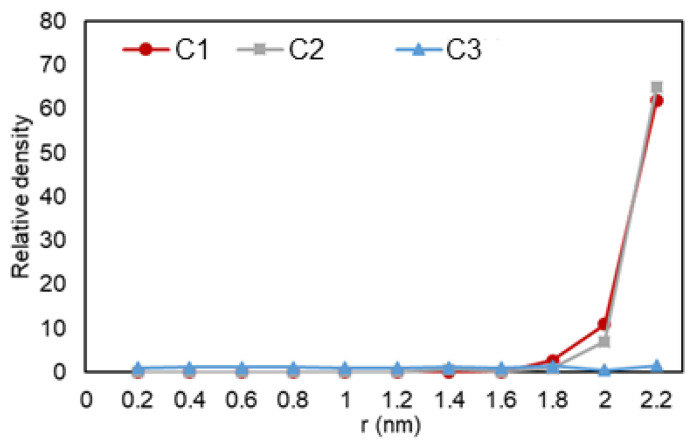
Ethane relative density (mass density inside the pore over the density of the phase in bulk condition) vs. radial position in C1 (gas phase in bulk), C2 (two-phase in bulk), and C3 (liquid phase in bulk) conditions.

Figure 8 shows how the heptane molecules are distributed along the radial distance from the pore center. Higher concentration of heptane molecules closer to the pore walls, which results in a denser phase, is interpreted as the adsorption of heptane molecules to the graphene wall. Since only nonpolar molecules are involved, the mechanism of adsorption can be again explained by the van der Waals interactions, induced dipole—induced dipole, between the hydrocarbon molecules and pore walls. Another interesting observation is a drop and a subsequent peak in density, which implies a second layer of adsorption for the liquid phase. Interestingly, here we did not recognize this peak for C2, while for ethane, we could observe peaks for both C2 and C3 cases. It might be that the two-phase condition here is more gas-like, as it is challenging to set the simulation pressure and temperature on certain values to achieve two-phase equilibrium considering that here we have only pure heptane. Figure 9 reveals the effect of nano-confinement on the distribution of heptane molecules in the nanopore. C1 and C2 conditions have similar pressures here, and they are the ones mostly affected when the pore was introduced to the system. This behavior can be explained by the lower pressures of C1 and C2, which result in less restriction to movement, so that molecules can interact with pore walls more freely compared to C3.

This set of simulations (single component cases) were important as an initial step to ensure that our simulation approach is appropriate to model mixture of different molecules behavior in different phases with and without nanopore confinement effects.

#### 3.1.3. Ethane/Heptane Mixture

**Figure 10:**
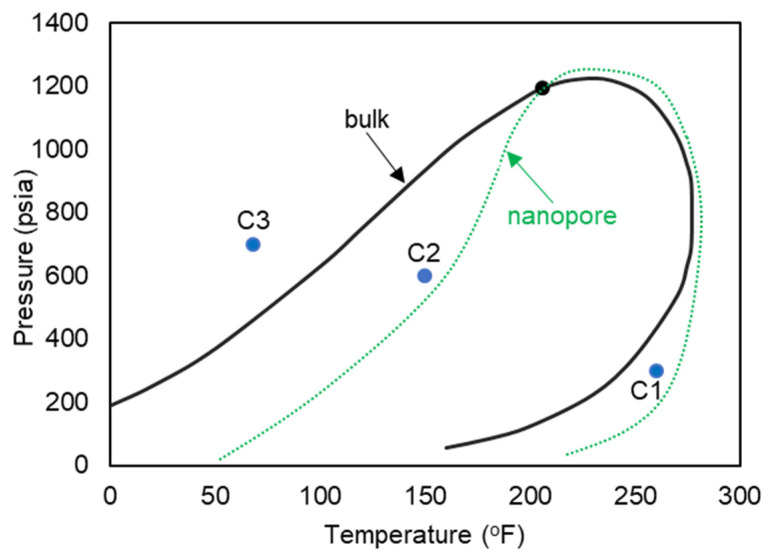
Pressure-temperature diagram of the ethane–heptane mixture.

Reservoir fluids are rarely composed of only one component; thus, in order to simulate a more realistic petroleum and natural gas fluid, a mixture of heptane and ethane is studied here. The mixture is composed of 70% ethane and 30% heptane from the total weight and the pressure–temperature diagram for this mixture at bulk is plotted using the Peng–Robinson equation of state [22] flash calculations, shown in Figure 10. These type of reservoir fluids are normally subjected to elevated temperatures and pressures, which induces cracking of heavier molecules into lighter ones. This then helps to explain the choice for a higher weight percent of ethane. However, since one of the goals of this study was to analyze phase transition in a mixture, it was necessary to introduce a heavier molecule, heptane, which is also normally encountered in unconventional reservoirs.

The three pressure and temperature conditions are shown with C1 (a gas phase in bulk), C2 (a two-phase in bulk), and C3 (a liquid phase in bulk) on the pressure–temperature diagram of Figure 10. The anticipated shifted phase envelope in the nanopore-confined space is also shown schematically on the phase diagram of Figure 10.

A total of six simulations were executed to observe the behavior of ethane–heptane mixture in C1, C2, and C3 conditions at the bulk and the confined space in the nanopore. The temperatures and pressures for C1, C2, and C3 conditions were acquired from a code created in our research group, which essentially implements the Peng–Robinson equation of state to analyze phase behavior of hydrocarbon mixtures.

**Figure 11:**
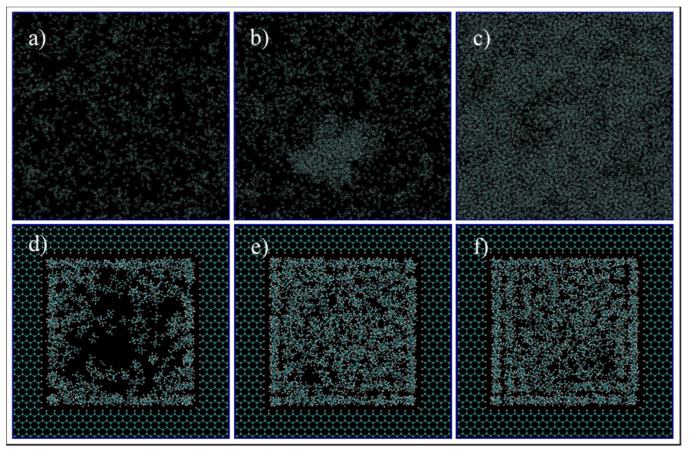
Ethane/Heptane mixture simulation in bulk (**a**–**c**) and pore medium (**d**–**f**) at C1 (**a**,**d**), C2 (**b**,**e**), and C3 (**c**,**f**) conditions.

Figure 11 exhibits images at the end of each simulation. For the gas phase in bulk, showed on the phase diagram of Figure 10 as C1, (Figure 11a,d, 260 °F, 300 psi), molecules are dispersed in the medium, and they move freely within the simulation box. However, when a confined space is introduced (Figure 11d), the molecules migrate inside the pore and accumulate mostly at the pore walls, with a higher concentration at the corners. The molecules near the walls have restricted movement with higher concentration of heptane, opposed to molecules at the center of the pore that are visually mostly composed of ethane. With these observations, it is possible to infer that molecules near the wall have a liquid like behavior while the ones at the center behave more like gas. This behavior can be translated to a dew-point pressure reduction for this temperature, so that the fluid is in the gas phase in the bulk, while the fluid behaves more like a gas–liquid mixture in the confined space of the nanopore. If these implications are valid, then the phase envelope of the ethane–heptane mixture may be shifted due to nanopore confinement. This shift is schematically shown on the phase diagram of Figure 10 with the green dashed curve. Similar results were also predicted by previous researchers through macroscale phase behavior analysis when they included nanopore confinement in their flash calculations through using equation of states [1,2,23,24]. For condition C3, (Figure 11c,f, 68 °F, 700 psi), the molecules are encountered in a high level of agglomeration, and their slight movements are restricted by the adjacent molecules, so both the bulk and the nanopore-confined ethane–heptane mixtures at C3 seems to have a liquid-like behavior. For condition C2, which results in the two-phase in bulk, (Figure 11b,e, 150 °F, 600 psi), two distinct behaviors for the molecules were observed: one was the molecules being dispersed and moving freely, which translates to a gas phase; and second was the molecules located on a condensed cluster moving relative to its adjacent molecules, which represents a liquid-phase cluster. For the nanopore simulation at C2, the behavior of the molecules is similar to the liquid phase. This observation may imply that the saturation pressure, a bubble-point pressure at this temperature, is suppressed in the confined space, so that the fluid behaves like a liquid phase in the nanopore. This observation is in agreement with the bubble-point pressure reduction in nano-confined space resulted from the inclusion of nanopore effects in flash calculations [23].

**Figure 12:**
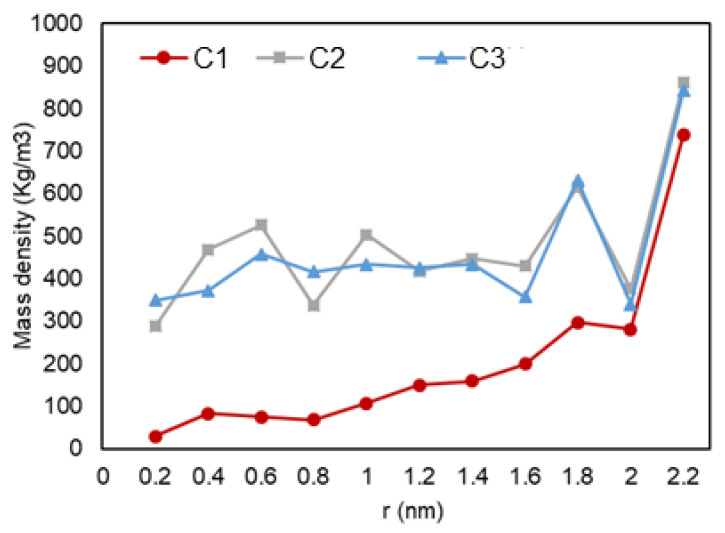
Mass density vs. radial position in C1 (gas phase in bulk), C2 (two-phase in bulk), and C3 (liquid phase in bulk) conditions.

Figure 12 shows a graph with the variation of mass density over the radial distance from to pore center. A similar trend is seen for all three setups, where a lower density is observed at the center of the pore and it reaches a peak when it is the closest to the pore wall. A second layer of adsorption is also recognized for C2 and C3 conditions (likely a liquid phase).

### 3.2. Hydrocarbon Mixture Composition Alteration

In previous sections, the effect of nano-confinement on density distribution and phase behavior has been identified. In a previous study, when the nanopore effect is considered, such as capillary pressure, the fluid-phase compositions inside nanopores and in bulk does differ, which in turn results in a different overall composition in the bulk compared to the nanopore, as a result of molecular diffusion [25]. In addition, it was observed that there is a tendency of an increase in heavier molecules’ composition in the nanopores (shale matrix) compared to the bulk (fracture) [25]. Recently, Zhao and Jin used a density functional theory to quantify the role of pore size distribution when nanopore confinement effects on the phase equilibrium are present. They observed that, even in the bulk, bubble-point pressure changed due to the confinement effects of the neighboring nanopores [26]. To further investigate the above-mentioned observations and to understand the reason for such phenomena, three simulations were performed, i.e., one for each temperature and pressure conditions of C1, C2, and C3, and the behavior of heptane, (the heavier component here) and ethane (lighter component here) were investigated through analyzing the hydrocarbon molecules distribution and overall compositions.

**Figure 13:**
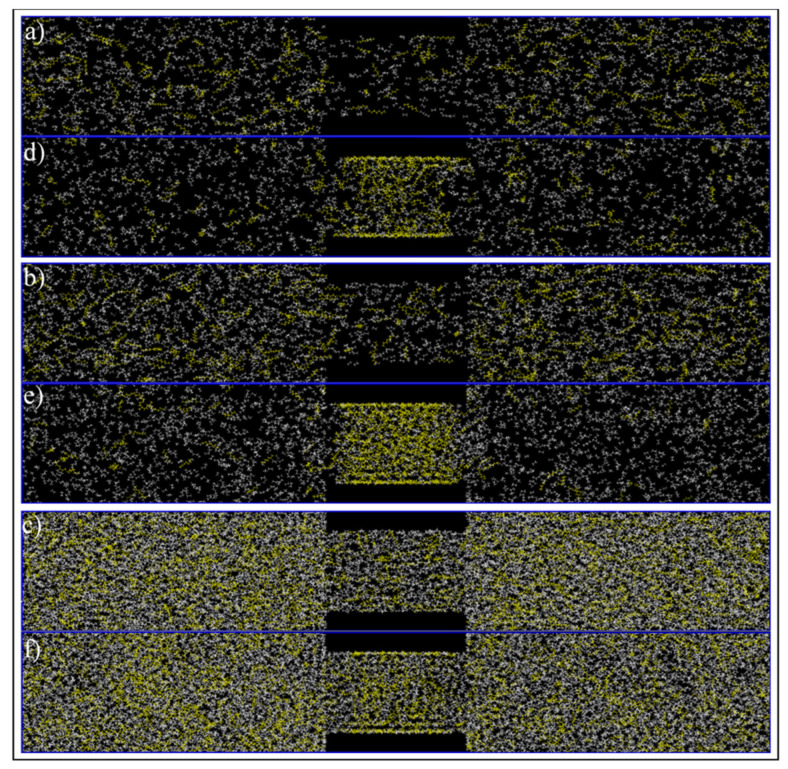
Initial (**a**–**c**) and final configuration (**c**–**e**) of ethane–heptane mixture at C1 (**a**,**d**), C2 (**b**,**e**) and C3 (**c**,**f**) conditions. Yellow and white colors represent heptane and ethane molecules, respectively.

Initially, the movement of hydrocarbon molecules was analyzed for the entire bulk-pore system. The initial and final configurations of the system is shown in Figure 13. For all cases, we observe an increase in the number of heptane molecules, yellow, inside the nanopore. In order to assess and confirm this visual observation, the heptane mass fraction in pore and bulk was acquired and the results can be seen in Table 1. Even though the molecules were placed randomly inside the pore, the initial mass fraction of heptane is close to the desired value of 30% for all locations and conditions. After the simulation is equilibrated, the mass fraction of heptane increases in the confined space of the nanopore for all three pressure and temperature conditions, while it decreases in the bulk. The heavier molecules migrate from the two large side pores (bulk) to the nanopore in the middle, and, eventually, the overall concentration of the mixture in the nanopore changes from its initial value of (70/30%) ethane–heptane to nearly 50/50% ethane–heptane (see Table 1 for the values). These results suggest that the overall composition of the nanopore system does change when the fluid interacts with the pore and the overall density of the mixture increases in the nanopore compared to the bulk. Although the trends observed here for composition change from bulk to pore and pore center to pore walls are present in all conditions, they are more predominant to the gas and two-phase conditions, especially to the latter one. For C2, heptane demonstrated the highest heptane mass composition increase in the pore when comparing the initial and final configurations, and it achieved the highest composition peak compared to other conditions at the pore walls. This behavior can be explained by the capillary effect, as surfaces are usually preferentially being wet by liquids over gas [27]. Since in C2, gas and liquid are present, the pore surface preferentially adsorbs the liquid phase, and since the liquid phase is mostly composed of heptane, the concentration of heptane molecules increases more efficiently than the other conditions. Our results here agree with our larger scale simulation results, stating that heavier molecules tend to migrate from the bulk (fracture) to the matrix (nanopore) [25]. This implies that, in the field scale, it is a challenge to recover heavier hydrocarbon molecules from the shale nanopores, as they have the tendency to remain in the confined space and not migrate to the fracture high permeability zone.

**Figure 14:**
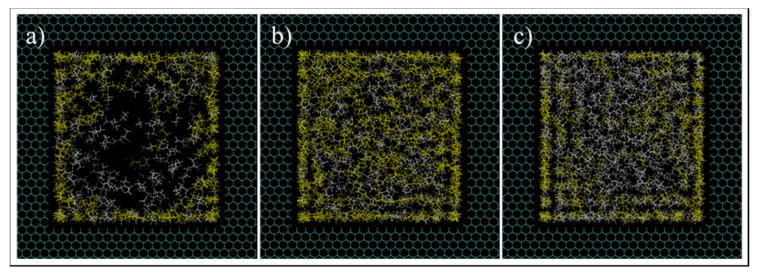
Images at the pore entrance of C1 (**a**), C2 (**b**), and C3 (**c**) conditions simulations. Yellow and white colors are heptane and ethane molecules, respectively.

**Figure 15:**
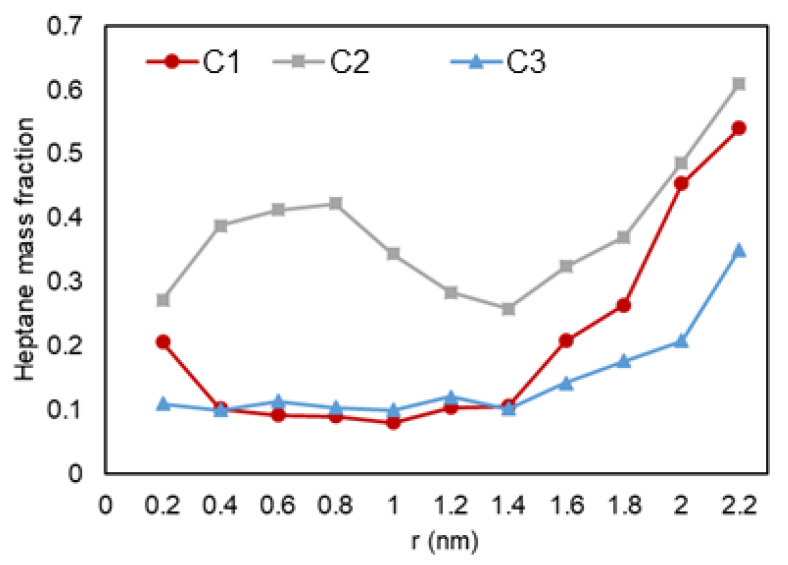
Heptane mass fraction (mass density inside the pore over the density of the molecules in bulk conditions) vs. radial position in C1 (gas phase in bulk), C2 (two-phase in bulk), and C3 (liquid phase in bulk) conditions.

Next, a detailed investigation of hydrocarbon molecules distribution inside the pore was performed. Images at the entrance of the pore can be seen in Figure 14. The radial mass fraction of heptane was calculated and plotted, as shown in Figure 15. As mentioned before, for all conditions, increased concentration of heptane in the pore is noted. For C1 and C3, heptane concentration increases from the center of the pore to the pore walls where a predominant yellow color is recognized. For C2 case, however, a relatively high heptane concentration spot near the pore center is recognized, which can imply the presence of a liquid droplet in the middle of the pore.

This observation that the fluid composition changes in all conditions, and that the mass fraction of the heavier component is larger in the smaller pores, can be explained by the van der Waals interaction between the pore and hydrocarbons. Since hydrocarbon and pore atoms can be considered non-polar molecules, their interactions are in the type of induced dipole—induced dipole. Since heptane molecule has a longer chain compared to ethane, it is easier for an induced dipole to form, and, consequently, these heavier molecules would have a higher interaction energy with the solid surface. This explains the movement of heptane molecules to the pore and their preference to be located closest to the pore walls.

Next, we quantitatively analyze the distribution of ethane and heptane in the graphite nanopore. As shown in Figure 15, in the nanopore that is in the middle, the heavier molecules, e.g., heptane (yellow color), tends to adsorb to the graphite wall while ethane molecules (white color) accumulate at the center (also shown in Figure 14). As mentioned before, heptane, which is here the heavier molecule, preferentially interacts/adsorbs on the surface of the wall since they have greater van der Waals forces and a longer chain. For C2, the increased concentration of heptane can indicate a trapped liquid droplet in the middle of the pore.

Mass transfer and composition alteration has been studied for a setup with the bulk phase in contact with a 5-nm cube-shaped pore so far. However, considering that shale rocks are heterogeneous in terms of pore size, it is relevant to analyze how these parameters behave when a 2-nm pore is introduced, which would represent two connected nanopores with different sizes. To do so, three simulations, with the same three phase conditions of the same ethane/heptane mixture, in a system with a bulk medium in contact with two 5-nm and 2-nm connected pores, were performed.

**Figure 16:**
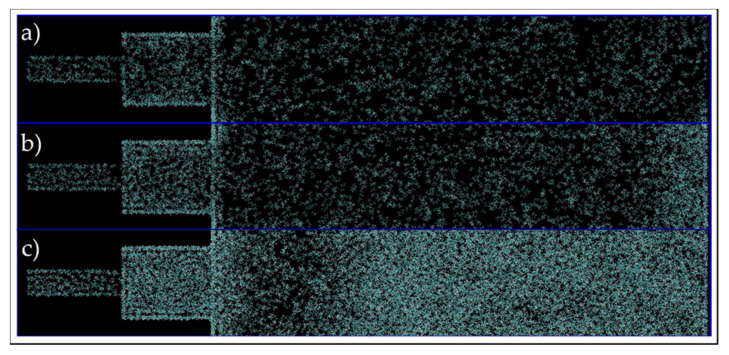
End of run images of the connected pore and bulk phase box, in C1 (**a**), C2 (**b**) and C3 (**c**) conditions simulations. Cyan and white colors represent carbon and hydrogen atoms, respectively.

**Figure 17:**
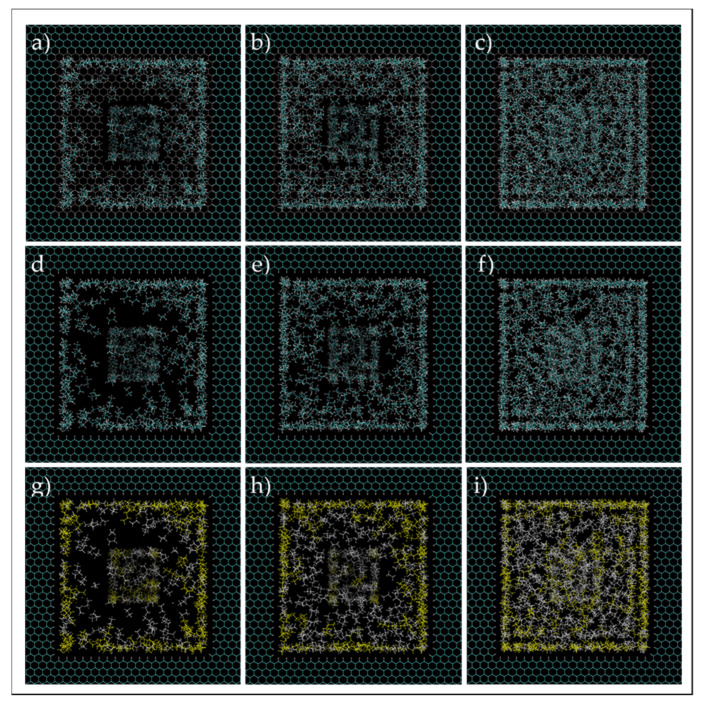
Ethane–heptane mixture simulation in two connected nanopores at C1 (**a**,**d**,**g**), C2 (**b**,**e**,**h**), C3 (**c**,**f**,**i**). Images (**d**–**f**) are the same as (**a**–**c**), but the 2-nm pore wall entrance is not shown in the former. Cyan and white spheres, for images at first and second rows, are carbon and hydrogen atoms, respectively. Yellow and white colors are heptane and ethane molecules, respectively, for images at the third row.

In order to have a perspective of phase and molecules behavior, the images of the system after 5 ns of simulation were investigated visually, which are shown in Figure 16 and Figure 17. For C1 (a,d,g) and C3 (c,f,i), the fluid molecules behave similarly to the single pore simulation, where there is a layer of high concentration of molecules near the walls of the 5-nm pore with a predominant presence of heptane molecules, and dispersed and agglomerated molecules in the middle of the pore, for C1 and C3, respectively. However, for C2 (b,e,h), even though it had the same behavior for molecules near the wall as the single pore simulation, the middle of the pore constitutes dispersed molecules with a high concentration of ethane, and, in contrast with the single pore case, a high concentration zone rich in heptane in the middle of the pore is not recognized. This observation can signify that when the 5-nm pore is in contact with a smaller pore, some heavier molecules tend to migrate to the 2-nm pore. Another observation is that the presence of nanopores also influences the distribution of ethane and heptane molecules in the bulk, whereas the bulk fluid is not homogenous anymore. This observation may be translated to changing the saturation pressure of the ethane–heptane mixture, not only in the nanopore, but also in the bulk adjacent to the nanoconfined space, which is in agreement with the conclusions of Zhao and Jin [26].

**Figure 18:**
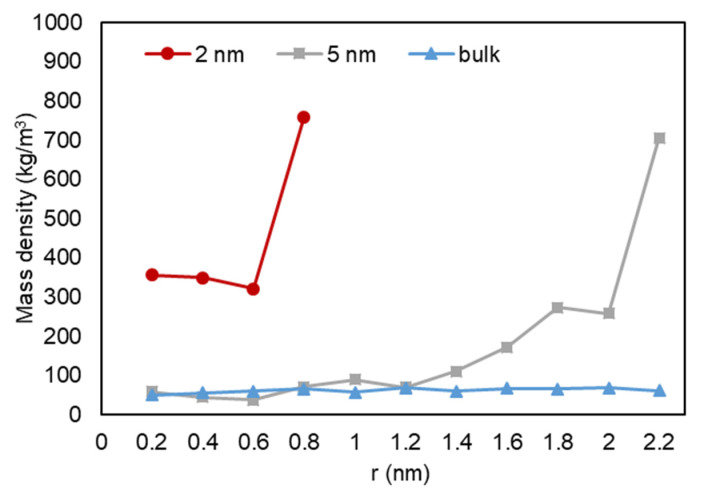
Mass density variation vs. radial position for C1.

**Figure 19:**
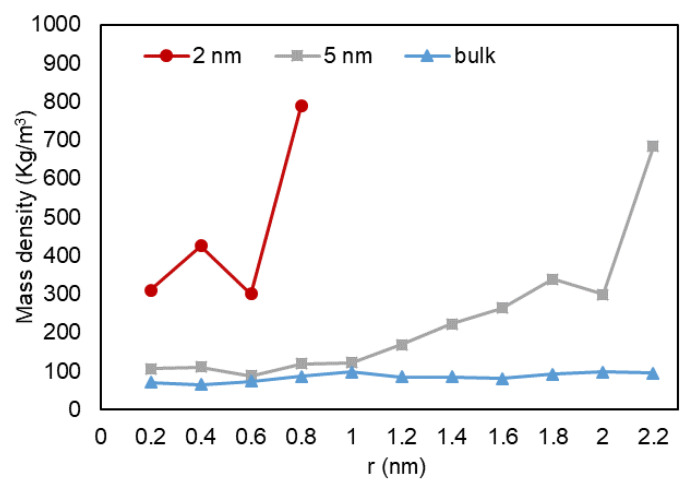
Mass density variation vs. radial position for C2.

**Figure 20:**
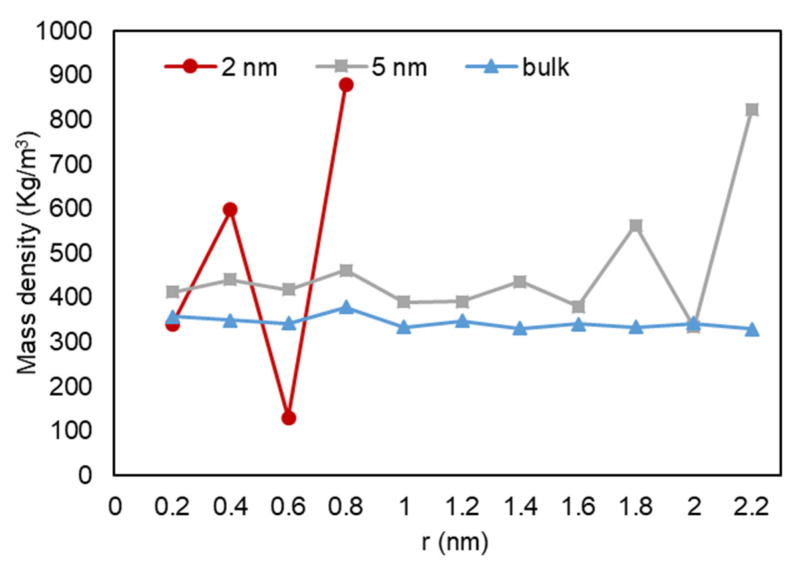
Mass density variation vs. radial position for C3.

A quantitative analysis was also performed to further investigate our previous visual observations. First, the mass density over the pore radius was analyzed and the output can be found in Figure 18, Figure 19 and Figure 20. For all cases, a similar trend for both pores are observed, where there is a peak in density closest to the pore walls, which can be translated to an adsorbed layer. In addition, both pores give a second peak, right after a depression in density, moving away from the pore walls, which can be interpreted as a second layer of adsorption. However, the mass density for both peaks is higher in the 2-nm pore compared to the 5-nm one. In addition, the density observed at the center of the C1 and C2 conditions is close to the liquid density. It can be concluded that the effect of nano-confinement on mass density becomes more significant with the pore size reduction.

**Figure 21:**
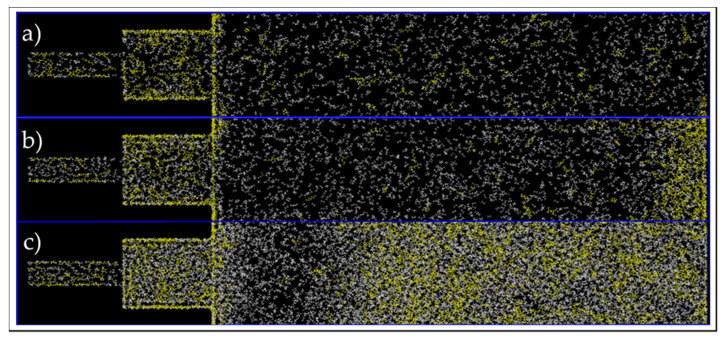
End of simulation images of the connected pores and bulk, at C1 (**a**), C2 (**b**), and C3 (**c**) conditions. Yellow and white colors represent heptane and ethane molecules, respectively.

Similar to the single 5-nm pore, the composition alteration of the 30/70 wt% ethane–heptane mixture was studied. Firstly, images at the end of simulations were investigated for a qualitatively analysis and they are shown in Figure 21. For all three conditions, there is a higher concentration of yellow color, which represents heptane molecules, inside the pores in comparison to this concentration in the bulk phase, where there is a predominant presence of white color and ethane molecules. In addition, for C2 and C3, shown in Figure 21b,c, respectively, it is recognized that the heptane molecules closer to the pores were the ones to enter the nanopores first, and consequently, an ethane-rich region is present adjacent to the entrance of the pore. Whereas, for C1, as shown in Figure 21a, the molecules are homogenously dispersed. This observation suggests that nano-confinement can lead to phase transition, in addition to compositional alteration and fluid property change, not only inside the pores, but also near the pore entrance in the bulk (in agreement with previous macro-scale studies [25,26]).

Next, the overall heptane mole fraction inside the pores and in bulk phase were computed and are shown in Table 2. As expected, the overall heptane mole fraction was higher inside the pores, when compared to bulk medium, in all cases. Furthermore, this parameter was even higher in the 2-nm pores compared to the 5-nm pore in all cases. This observation is in concordance with the increase in mass density of the pores, which reinforces the idea that the nano-confinement effect in compositional alteration increases with the decrease in the pore size.

## 4. Conclusions

In this study, molecular dynamics simulation is used to investigate the effect of nanopore confinement on phase behavior, density, and composition of hydrocarbon fluids. The density profile and concentration distribution of a 70–30 weight % ethane–heptane mixture in multiple sets of connected graphite nano-pores and bulk conditions are analyzed. Three different pressure and temperature sets resulting in gas, gas–liquid, and liquid phases are considered. For all the cases, hydrocarbon molecules tend to adsorb to the pore surface, so that the fluid density is the highest near the solid pore surface. The other observation is that heptane molecules get adsorbed to the surface more effectively compared to ethane. The preferential adsorption of heptane results in an increased heptane concentration in the smallest pores because of the lowest volume/surface ratio.

The highlights of this research are:

- Hydrocarbon density profiles are not homogenous in graphene nanopores with square cross sections. In most of the cases, two density peaks are observed near the pore surface. These peaks are characterized as adsorption layers. For the smallest pore (2 nm), the entire hydrocarbon molecules are present as an adsorbed phase.
- The phase behavior of the ethane–heptane mixture is different in an oil-wet graphene nanopore compared to the bulk. Bubble-point pressure is likely suppressed in the nanopore-confined space, but we believe that more case studies are needed to generalize this idea.
- Overall composition of the hydrocarbon mixture in a nanopore that is connected to the bulk space is different from that in the bulk. The overall compositions in two different nanopores with different sizes are also different.
- The concentration of the heavier components is higher in the smaller pores.
- The concentration gradient may be the driving force for molecular diffusion. Considering that permeability is very low for shale and tight rocks, diffusion is likely the dominant mass transfer mechanism.
- For nanopores with pore size distribution in shale, overall composition of reservoir fluid is not homogenous, and modifications should be made to reduce the error when calculating original hydrocarbon in place.
- In fractured reservoirs, the heavier hydrocarbons tend to be in the nanopores, while lighter and more volatile molecules are likely to move to the fracture and be recovered easier.

## Figures and Tables

**Table 1:** Initial and final heptane mass fraction in bulk and inside the nanopore-confined space.

Condition	Bulk—Initial C_7_H_16_ Mass Fraction	Nanopore—Initial C_7_H_16_ Mass Fraction	Bulk—Final C_7_H_16_ Mass Fraction	Nanopore—Final C_7_H_16_ Mass Fraction
C1 (gas in bulk)	0.3206	0.3447	0.2411	0.5087
C2 (two-phase in bulk)	0.3052	0.2858	0.1301	0.6991
C3 (liquid in bulk)	0.3147	0.2658	0.3068	0.3967

**Table 2:** Initial and final heptane mass fraction in 2-nm and 5-nm pores and bulk mediums.

Domain	Initial	C1—Final	C2—Final	C3—Final
2-nm Pore	0.3	0.48	0.34	0.34
5-nm Pore	0.3	0.37	0.33	0.31
Bulk	0.3	0.21	0.14	0.28

## Data Availability

The data presented in this study is available on demand.
